# Genetic and epigenetic dependencies in colorectal cancer development

**DOI:** 10.1093/gastro/goac035

**Published:** 2022-08-12

**Authors:** Sehej Parmar, Hariharan Easwaran

**Affiliations:** Cancer Genetics and Epigenetics, Oncology, Sidney Kimmel Comprehensive Cancer Center, Johns Hopkins University School of Medicine, Baltimore, MD, USA; Cancer Genetics and Epigenetics, Oncology, Sidney Kimmel Comprehensive Cancer Center, Johns Hopkins University School of Medicine, Baltimore, MD, USA

**Keywords:** colorectal cancer, epigenetics, aging, classification, subtyping, DNA methylation, consensus molecular subtypes

## Abstract

Recent studies have mapped key genetic changes in colorectal cancer (CRC) that impact important pathways contributing to the multistep models for CRC initiation and development. In parallel with genetic changes, normal and cancer tissues harbor epigenetic alterations impacting regulation of critical genes that have been shown to play profound roles in the tumor initiation. Cumulatively, these molecular changes are only loosely associated with heterogenous transcriptional programs, reflecting the heterogeneity in the various CRC molecular subtypes and the paths to CRC development. Studies from mapping molecular alterations in early CRC lesions and use of experimental models suggest that the intricate dependencies of various genetic and epigenetic hits shape the early development of CRC via different pathways and its manifestation into various CRC subtypes. We highlight the dependency of epigenetic and genetic changes in driving CRC development and discuss factors affecting epigenetic alterations over time and, by extension, risk for cancer.

## Introduction

Colorectal cancer (CRC), which involves cancers occurring in the colon and rectum, is the third most prevalent cancer by incidence and mortality worldwide. In fact, it is estimated that by 2030 there will be globally >2.2 million new cases and 1.1 million deaths due to CRC [1]. CRC is a heterogenous group of tumors in the colon and rectum that can be sub-grouped based on distinct molecular patterns, including pathological parameters, genomic alterations, and gene-expression patterns. This heterogeneity in the molecular patterns of CRC further reflects differences in the molecular evolution of CRC from the early stages of tumor initiation to its clinical presentation, including different molecular drivers of carcinogenesis, routes to tumor progression, factors contributing to the risk of cancer development, and potentially even cell of origin. Recent genome-wide sequencing projects and development of experimental models have revealed the importance of the interaction of genetic and epigenetic alterations in the early stages of CRC molecular evolution [2–6]. This review will discuss recent advances in the molecular genetics of early CRC evolution and examine the role early epigenetic changes may have in creating the appropriate gene-expression profile for genetic driver mutations to promote CRC tumor initiation and progression. Within this context, we strive to answer the following questions: Which key physiological and environmental factors contribute to epigenetic changes? How do these epigenetic changes play a role in tumor initiation and increasing cancer risk by genetic driver mutations?

### Molecular heterogeneity of CRC

CRC carcinogenesis occurs due to the progressive accumulation of different combinations of molecular changes in the form of genetic and epigenetic insults during aging that inactivate tumor suppressor genes and activate proto-oncogenes [7, 8]. Classifying CRC based on these molecular changes, which is still an ongoing process, has immense value for understanding essential events in CRC evolution and their clinical implications. Developments in molecular and genomic technologies have continually refined the classification of CRC into various subtypes over the years. Recent integrated molecular characterizations of CRC, such as The Cancer Genome Atlas (TCGA) project, have revealed important relationships among the genetic, epigenetic, and transcriptional changes [9]. Earlier molecular classifications were primarily based on genetic and epigenetic parameters, while newer classifications have also incorporated transcriptional signatures for more robust clinical implications [10–12]. Below we discuss the relationships between the molecular features underlying both the conventional classification and the recent gene-expression-based classification, which serve in further understanding the genetic and epigenetic basis for CRC evolution.

### Conventional classification

CRC is primarily classified based on the following genetic and epigenetic molecular features, which are important determinants of the alternate routes of genomic instability in CRC development.

### Chromosomal instability

The chromosomal instability (CIN) subtype is the most prevalent feature accounting for 80%–85% of all CRC cases and is characterized by sporadic genomic instability. It is defined by abnormalities in either the chromosomal structure resulting in loss of heterozygosity (LOH) or somatic copy number alterations (SCNA; aneuploidy or polyploidy) associated with a specific set of tumor suppressor and proto-oncogene loci [13–15]. A hallmark of 70%–80% of CIN cases is loss of the tumor suppressor and Wnt pathway negative regulator *APC*, which occurs by sporadic mutation in one allele of *APC* followed by loss of the other *APC* allele due to flaws in chromosome segregation [16, 17]. Other genes that are typically affected include *TP53*, *KRAS*, and *PIK3CA*. LOH in chromosome 18q containing tumor suppressor genes *SMAD2*, *SMAD4*, and *DCC* is also common as these genes are transcriptional mediators of the transforming growth factor (TGF)-β signaling pathway, which regulates cell growth, differentiation, and apoptosis, and promotes MYC activation [9, 16, 18]. CRC with CIN is more frequently observed in the distal colon [17].

### Microsatellite instability

CRC classified as microsatellite instability (MSI) form the other major type of genomic instability aside from CIN. MSI develops due to inactivation of the mismatch repair genes and accounts for 15%–20% of CRC. Twenty percent of MSI cases are hereditary and develop due to germline mutations in the mismatch repair genes, specifically *MLH1*, *MSH2*, *MSH6*, and *PMS2*. The remaining 80% of these cases are sporadic and develop due to spontaneous epigenetic silencing of the *MLH1* gene by promoter hypermethylation in the CpG-island methylator phenotype (CIMP) [14, 19]. Rather than affecting whole or parts of chromosomes, MSI is defined by a high frequency of replication errors, specifically an accumulation of insertions and/or deletions, in repetitive mononucleotide and dinucleotide DNA sequences referred to as microsatellites in exon regions of tumor suppressor genes, resulting in loss of function hypermutation [18]. MSI tumors have been subclassified into three groups based on the number of microsatellites associated: (i) high MSI (MSI-H), (ii) low MSI (MSI-L), and (iii) microsatellite stable (MSS). Frequently targeted genes for mutation in MSI tumors include *BRAF* (which is mutated in >8% of all CRC cases), *ACVR2A*, *TGFBR2*, *MSH3*, and *MSH6* along with Wnt pathway regulators *RNF43*, *RNF213*, and *ZNRF3* [20, 21]. LOH and mutations in *APC*, *TP53*, and *KRAS* are less frequent in MSI tumors than in CIN tumors, with loss of APC dropping from 80% down to ∼50% in MSI cases [9]. CRC with MSI status is more frequently observed in the proximal colon [17]. In contrast to the MSI tumors, CIN tumors are non-hypermutated and MSS.

A more recent and less well-studied class of MSI that is different from the classical MSI is the elevated microsatellite alterations at selected tetranucleotide repeats (EMAST), which occurs due to mismatch repair dysfunction resulting from loss of function of MSH3 [22]. Herein, MSH3 loss of function is caused by nuclear to cytoplasmic export of MSH3 in response to oxidative stress and pro-inflammatory cytokine interleukin (IL)-6 [23]. Unlike MSI due to MLH1 loss, which occurs in ∼15% of all CRC cases, EMAST is observed to be prevalent in 60% of CRC [24]. MSH3 dysfunction can also contribute to defects in double strand DNA repair by homologous-recombination, leading to the CIN phenotype [25]. Thus, microenvironmental changes, such as those that cause oxidative stress or inflammatory exposure of colon cells, may lead to this alternate form of MSI, i.e. EMAST, which may play a role in genetic events that lead to a substantial number of CIN CRC.

### CpG-island methylator phenotype

CIMP tumors are characterized by a very high frequency of promoter gene methylation [26, 27]. In general, ∼50% of gene promoters in humans harbor 200–800 base-pair stretches of nucleotides called CpG-islands that are unusually enriched with the dinucleotide sequence containing cytosine followed by guanine (termed CpG-residues). CpG-islands have important roles in the regulation of gene expression, and methylation of the CpG-islands in promoters causes silencing of these genes [26]. Widespread hypermethylation involves methylation of the promoter regions of many tumor suppressor genes leading to their epigenetic silencing and is present in 20%–30% of CRC cases, both MSI and CIN [28, 29]. CIMP classification of CRC represents the spectrum of CRC with differing frequency of hypermethylated CpG-island promoters [30, 31]. Based on the degree of methylation at a group of genes/loci, clinically cancers with the highest frequency of hypermethylation of these marker loci are classified into CIMP-high (CIMP-H), those with intermediate frequency are classified into CIMP-low (CIMP-L), and those lacking hypermethylation of the marker loci are classified as CIMP-negative (CIMP-). In general, the CIMP-H cancers have variably been associated with poor outcomes [32, 33] with the CIMP-H being a negative indicator of overall survival, whereas MSI-H is a positive indicator [34, 35]. Recent pooled analysis suggests that CIMP-H CRC harboring *BRAF* mutation but with MSI-L status have the highest disease-associated mortality [36]. CIMP-H is prevalent in tumors in the proximal colon with MSI-H and *BRAF* mutation, whereas CIMP-L tends to occur in the context of *KRAS* mutations in the distal colon; however, there are also wide heterogeneities in these co-occurrences and their corresponding impact on disease outcomes as CIMP-H can also occur in CIN (MSI-L/MSS) tumors in the proximal colon with higher frequency of *BRAF* mutations, which tends to have worse survival [36, 37]. These incidence patterns of CIMP in relation to other molecular features suggest that CIMP is an important and early premalignant pathological pathway particularly involved in *BRAF*-induced CRC development.

### Transcriptional subtypes and their relation to conventional subtypes

**Figure 1.**
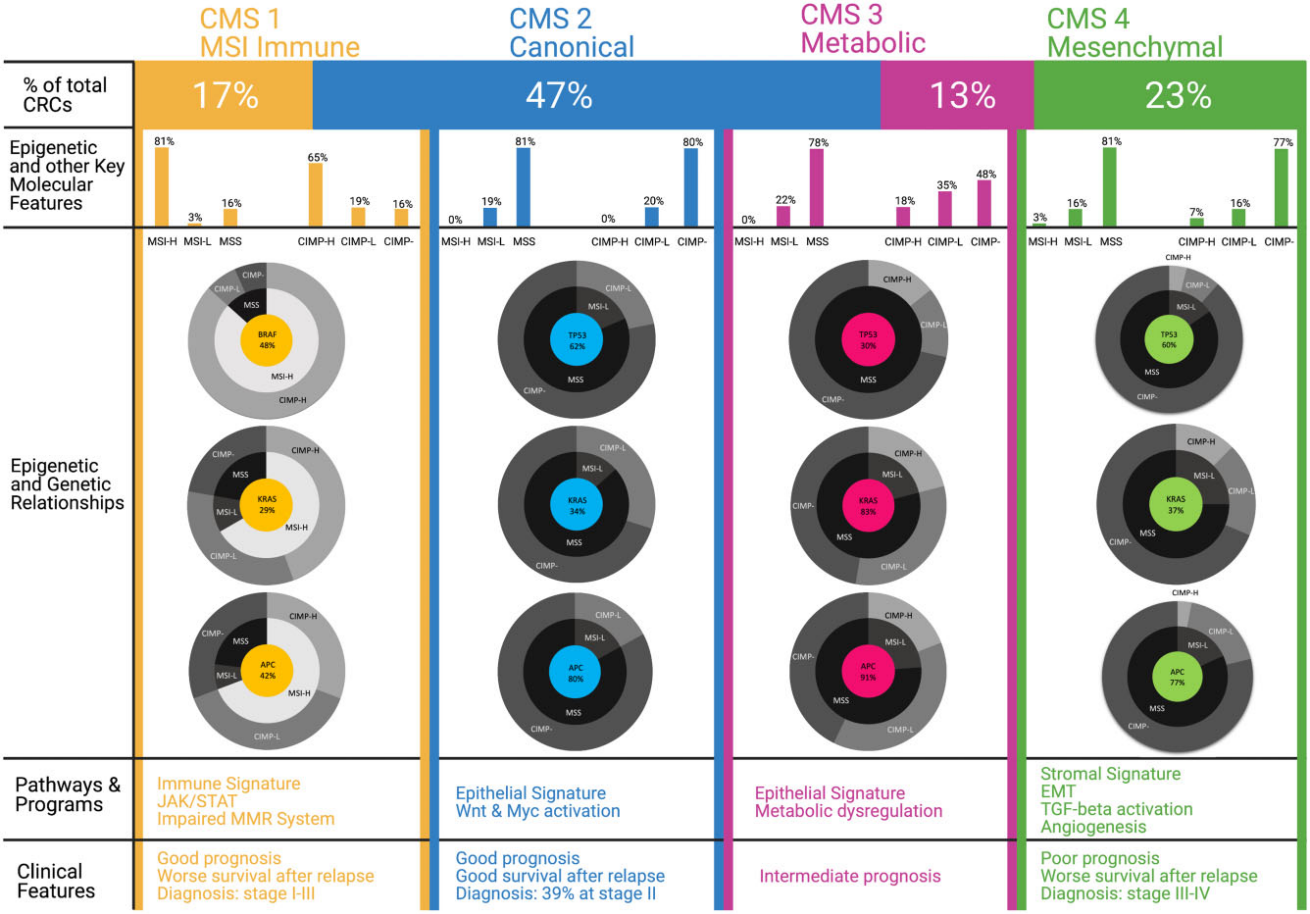
Epigenetic features of the various CRC CMS subtypes. Mutation frequencies of key genes (*APC*, *KRAS*, *BRAF*, *TP53*) and the MSI status and CIMP status of CRC samples in the TCGA dataset (185 samples) available on cBioPortal is related to the consensus molecular subtypes (CMS). Each transcriptional subtype shows distinct molecular and clinical features within the CMS classification. CMS1 (MSI immune) cancers show a high MSI and CIMP status, *BRAF* mutations, diffuse immune infiltration, and good prognosis but worse survival after relapse. On the other hand, CMS2–4 cancers show an MSS and CIMP-low/negative status. CMS2 (canonical) cancers display a high level of CIN, loss of *APC* and *TP53* mutations, and activation of the Wnt and MYC signaling pathways. CMS3 (metabolic) cancers are characterized by their low level of CIN, overrepresentation of *KRAS* mutations, and dysregulation of metabolic pathways, including carbohydrate and fatty acid oxidation. Similar to CMS2, CMS4 (mesenchymal) cancers display a high level of CIN, loss of *APC* and *TP53* mutations, and diffuse stromal infiltration along with worse survival overall and after relapse. Figure generated using BioRender. MSI, microsatellite instability; MSS, microsatellite stable; CIMP, CpG-island methylator phenotype; CIN, chromosomal instability; MMR, mismatch repair; EMT, epithelial-to-mesenchymal transition.

The conventional classification of CRC discussed above provides the critical reference point for understanding molecular drivers of CRC and guiding therapeutic and response dynamics. However, it does not encompass the diversity of CRC phenotypes and contribution of transcriptional landscape to these molecular genetic phenotypes. An integrated transcriptome-based classification using approaches from six independent studies have classified CRC into the consensus molecular subtypes (CMS), which has helped better understand biological features and molecular properties of the various CRC subtypes [38]. Based on the CMS clustering approach, 80%–90% of CRC falls into one of the four major transcriptional subgroups termed CMS1–4 and the remaining are heterogeneous cases exhibiting “mixed or indeterminate” gene-expression patterns with varying features of these subtypes [21, 38, 39]. The most striking outcome of the CMS classification is the separation of CRC into subtypes representing the following major molecular pathways that also associate with the molecular genetics and epigenetic phenotypes as well as the site of colon at which the CRC originates [40, 41]: (1) CMS1 represents the MSI immune subtype characterized by diffuse immune infiltrate with concomitant immune evasion signatures. (2) CMS2, also known as the canonical subtype, has a strong epithelial signature with activation of Wnt and MYC targets. (3) CMS3 is characterized by deregulation of metabolic signature pathways. (4) CMS4 is characterized by signatures of increased epithelial-to-mesenchymal transition (EMT), activation of TGF-β signaling, extracellular matrix remodeling, angiogenesis, and the complement-mediated inflammatory system (Figure 1).

**Figure 2.**
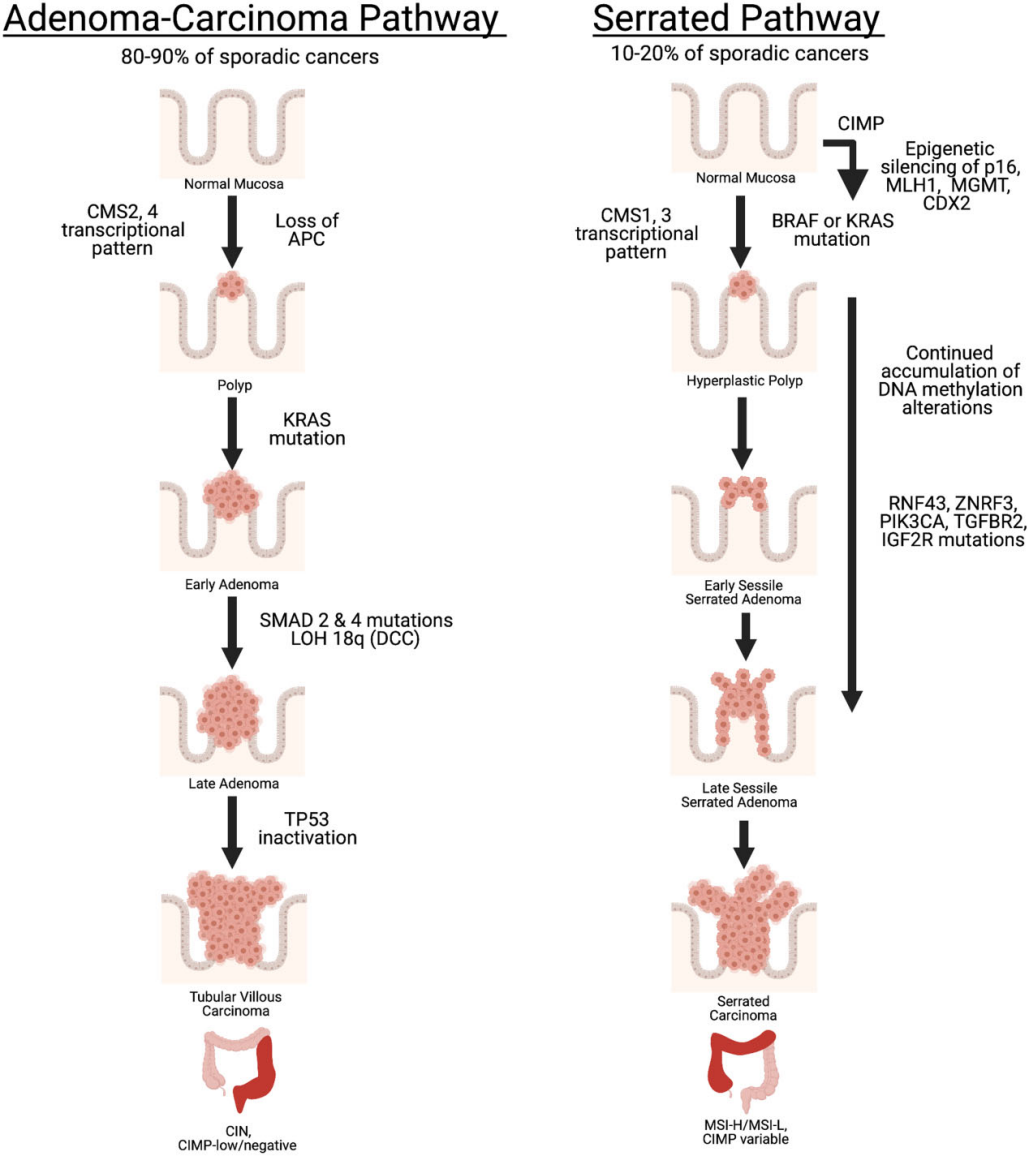
Molecular dependencies of adenoma–carcinoma and serrated pathways. In the classical adenoma–carcinoma pathway, activation of both the Wnt pathway via *APC* loss and MEK–ERK pathway via the *KRAS* oncogene followed by tumor suppressor inactivation of *SMAD4* and *TP53* develops microsatellite stable (MSS), CpG-island methylator phenotype (CIMP)-low/negative cancers with chromosomal instability (CIN). In the serrated pathway, activation of the MEK–ERK pathway occurs via the *KRAS* or *BRAF* oncogenes in parallel with CIMP-high related DNA methylation accumulation. This involves methylation of important genes such as *CDKN2A/p16*, *MLH1*, and *CDX2*. Methylation at the *MLH1* promoter results in high microsatellite instability (MSI-H) cancers, while cases lacking *MLH1* methylation result in low microsatellite instability (MSI-L) cancers. Other DNA repair genes, such as *MGMT*, also get affected by promoter methylation. Mutations in various genes most likely occur later during progression of the serrated lesions, leading to dysregulation of the major cancer pathways, including due to inactivation of the RNF43–ZNRF3 axis for Wnt pathway activation. Consensus molecular subtypes (CMS) expression phenotypes may be an early event occurring at the early lesion stage and may have important roles in the path to tumor development. Figure generated using BioRender.

Although the CMS subtypes provide a transcriptional basis for the biological features of CRC and relate it to outcomes, it paints a complex picture regarding the conventional molecular subtypes mentioned above, with only CMS1 showing the highest association with the conventional classification in that these tumors are highly enriched for the CIMP-H, MSI-H, and low CIN hypermutator cases (Figure 1). The remaining CRC cases showing CIN and MSS are distributed among the CMS2–4 subtypes. Reflecting the heterogeneous nature of CRC, key genetic mutations that are known drivers of CRC do not show distinct association with the CMS subtypes. The only exceptions to this finding are CRC with mutations in *BRAF* or *KRAS*, which are distributed among CMS1 and CMS3, respectively (Figure 1). Such diversity in a transcriptional landscape that is very loosely associated with genetic mutations most likely hints at different cells of origin, which in turn depends on the epigenetic state of the cells, or same cells of origin but with different acquired epigenetic alterations in response to environmental cues. CRC with the *BRAF* and *KRAS* mutations, derived from the serrated or conventional adenoma-to-carcinoma pathways (discussed further in detail later), are also represented by distinct epigenetic subtypes that may represent basic differences in transcriptional programs involved in these cancers. The gene-expression signatures of *BRAF-* and *KRAS*-mutated tumors typifying the CMS subtypes have been observed in adenomas, the precursors of CRC, indicating that these gene-expression patterns are early changes during CRC evolution that occur before acquisition of all of the relevant cancer-driver mutations [32, 42] (Figure 2). These observations in early adenomas are suggestive again that a significant component of the transcriptional programs contributing to the CMS types may be driven more so by the cell of origin or the epigenetic state of the cells that give rise to tumor clones than by the driver mutations occurring later during adenoma-to-carcinoma conversion.

The distinction of CRC into different CMS expression types with no underlying mutational drivers, first, strengthens observations made over the years that CRC is a very heterogenous group of tumors and, second, indicates that although CRC is initiated by common driver events, the manifestation of the expression phenotypes may be subject to various other factors, such as the cell of origin, tissue location, and the role of factors epigenetically predisposing to tumorigenesis, such as aging, inflammation, environmental exposures, metabolic factors, and microbiome [40]. Gaining a clear understanding of the drivers of CMS expression phenotypes warrants further analyses by inclusion of these various other factors.

### Genetic and epigenetic contributions to the major pathways of CRC development

Genome-wide exome sequencing studies have revealed a plethora of mutations and epigenetic alterations that are involved in genome maintenance along with the Wnt, MEK–ERK, PI3K, TGF-β, and p53 pathways to be the primary mediators of CRC development [9, 43]. A key insight this model provides is to view tumor initiation as a consequence of the dysregulation of a limited set of these four to five pathways. As discussed in detail below, epigenetic mediated gene silencing plays parallel, if not similar, roles in dysregulation of these pathways and there is a close interdependency between the genetic and epigenetic mechanisms in driving CRC initiation. In addition, the differences in the spectrum of mutations and epigenetic alterations for CRC initiation at various sites along the colon axis starkly reflect different dependencies on disruption of these above pathways (Figure 2). These alterations are early events also observed in the adenomas, and thus closely reflect the major routes to CRC development described below (Figure 2).

### Classical adenoma–carcinoma pathway

The majority (50%–70%) of CRC cases arise via the classical adenoma–carcinoma pathway that begins as an adenomatous precursor lesion protruding from the epithelial lining [8]. The incidence and risk for advanced adenomas and carcinomas increase with age, with ∼25% of men in the USA by age 50 years showing adenomas [44–46]. The incidence rate of adenomas in women is 2- to 3-fold lower than that of men [44]. Approximately 10% of these adenomas in both men and women progress to carcinomas when unchecked, indicating various tissue and immune-microenvironment-based controls that prevent development of carcinomas [47]. The pathological and molecular features of a sporadically occurring adenoma–carcinoma pathway closely mimic the CRC arising in the familial adenomatous polyposis syndrome. In most cases of the adenoma–carcinoma sequence, the development of aberrant crypt foci in the initial stages of CRC development involves unregulated activation of the Wnt pathway via an inactivating *APC* mutation. Next, the founder cells acquire further mutations in *KRAS*, *SMAD4*, and *TP53* [9, 48, 49] followed by copy number alterations and additional epigenetic changes during development of a benign adenomatous polyp to transition to an advanced premalignant polyp with high-grade dysplastic foci and finally to an invasive carcinoma [50] (Figure 2). A cornerstone of the adenoma–carcinoma pathway is the development of early Wnt independency combined with mutations activating MEK–ERK signaling and inactivating cell-cycle check-point pathways [51]. Wnt independency occurs mainly due to *APC* mutation, which is found to be inactivated in 75% of CRC cases arising via the conventional adenoma–carcinoma pathway [52–54], and the subsequent dysregulation of MEK–ERK signaling and cell-cycle check points is primarily due to the high likelihood of *APC* mutations to co-occur with *KRAS* mutations and/or *TP53* mutations. When this co-occurrence is present during progression of the adenoma–carcinoma sequence, *APC* tends to be the primary mutation and is followed by *KRAS* mutation, *TP53* mutation, or both [55] (Figure 2). The importance of Wnt activation in this progression is exemplified using genetically engineered mouse models which show that sustained activation of the Wnt pathway by loss of *Apc* function is a key driver of the tumorigenic property as restoration of *Apc* completely reverses the tumor phenotype by reestablishing normal colon stem-cell differentiation [56]. However, in human CRC, although Wnt activation by *APC* mutations sufficiently activates Wnt signaling [57], multiple other mutations in the Wnt pathway are observed in the adenomas in both familial adenomatous polyposis patients [58] and sporadic adenomas [59, 60], and the resulting sporadic carcinomas [9]. These mutations include important Wnt pathway genes, such as *LRP1B*, *SOX9*, *FAT4*, *TCF7L2*, *FBXW7*, *ARID1A*, *CTNNB1*, and *AXIN2* (Table 1). Thus, these additional genetic defects in the Wnt pathway suggests dependencies on mechanisms for sustained activation of the Wnt pathway in addition to that from loss of APC function.

**Table 1.** Summary of the various gene mutations in CRC characterized in the TCGA [9] and DFCI [175] sequencing projects

Gene	Sample size (#)	Proximal colon cancer (%)	Distal colon cancer (%)	Rectal cancer (%)	Pathway	Function
MLH1 (P)	25	59.6	25.2	15.2	DNA mismatch repair	MMR after DNA replication; forms heterodimer with PMS2
MLH3 (P)	22	68.2	14.4	17.4	DNA mismatch repair	MMR after DNA replication; competes against PMS2 to from heterodimer with MLH1
MSH2 (P)	12	48.8	23.2	28.1	DNA mismatch repair	MMR after DNA replication; binds to MSH6 or MSH3 to form heterodimer
MSH3 (P)	20	54.3	36.8	8.9	DNA mismatch repair	MMR after DNA replication; binds with MSH2 to form heterodimer that recognizes insertion-deletion loops
MSH6 (P)	27	62.5	16.9	20.5	DNA mismatch repair	MMR after DNA replication; binds MSH2 to form heterodimer
PMS1 (P)	16	69.5	30.5	0	DNA mismatch repair	MMR after DNA replication; forms heterodimer with MLH1
PMS2 (P)	18	49.6	31.4	19.0	DNA mismatch repair	MMR after DNA replication; forms heterodimer with MLH1
POLE (P)	47	49.2	36.2	14.6	DNA mismatch repair	Involved in chromosomal DNA replication, recombination, and DNA repair via base and nuclear excision pathways
APC	361	29.7	32.0	38.3	Wnt signaling	Tumor suppressor; regulator of Wnt pathway and involved in cell cycle and cell adhesion
AXIN1	23	63.6	20.1	16.3	Wnt signaling	Tumor suppressor; component of β-catenin destruction complex
AXIN2	49	55.1	26.9	18.1	Wnt signaling	Tumor suppressor; component of β-catenin destruction complex
CTNNB1	36	49.6	31.4	19.0	Wnt signaling	Oncogene; makes protein product β-catenin ligand of Wnt pathway
TCF7L2	44	34.8	37.3	27.9	Wnt signaling	Tumor suppressor; TF for many genes by altering the chromatin structure around those genes, suppresses transcription of CTNBB1
ARID1A (P)	67	43.6	34.4	22.0	Wnt signaling	Tumor suppressor; TF for many genes by altering the chromatin structure around those genes, suppresses transcription of CTNBB1
FBXW7	86	35.5	30.7	33.7	Wnt signaling	Tumor suppressor; involved in the ubiquitination and degradation of the cell-cycle regulators
RNF213 (P)	80	59.3	22.5	18.2	Wnt signaling	Tumor suppressor; ubiquitin ligase that acts on frizzled receptors in Wnt pathway
RNF43 (P)	72	81.7	9.6	8.7	Wnt signaling	Tumor suppressor; ubiquitin ligase that acts on frizzled receptors in Wnt pathway
ZNRF3 (P)	38	68.6	21.1	10.3	Wnt signaling	Tumor suppressor; ubiquitin ligase that acts on frizzled receptors in Wnt pathway
SOX9	62	41.2	25.4	33.4	Wnt signaling	Tumor suppressor; TF for intestinal stem-cell differentiation; promotes ubiquitination and degradation of β-catenin
FAM123B/WTX	NA	NA	NA	NA	Wnt signaling	Tumor suppressor; promotes ubiquitination and degradation of β-catenin, stabilizes Axin2
KLF5	14	38.9	27.6	33.5	Wnt signaling	Oncogene; plays a critical role in β-catenin activation by increasing interaction with TCF4
TGFBR1 (P)	8	42.0	0	58.0	TGF-β signaling	Tumor suppressor; component of TGF-β receptor
TGFBR2 (P)	29	60.3	20.8	18.8	TGF-β signaling	Tumor suppressor; component of TGF-β receptor
SMAD2	32	47.4	21.4	31.2	TGF-β signaling	Tumor suppressor; downstream signaling molecule for TGF-β signaling, when phosphorylated forms SMAD2-SMAD4 heterodimer to modify transcription of TGF-β target genes
SMAD3 (P)	21	39.2	30.9	29.9	TGF-β signaling	Tumor suppressor; downstream signaling molecule for TGF-β signaling, when phosphorylated forms SMAD3-SMAD4 heterodimer to modify transcription of TGF-β target genes
SMAD4	73	39.8	34.1	26.1	TGF-β signaling	Tumor suppressor; downstream signaling molecule for TGF-β signaling, when phosphorylated forms SMAD2/3-SMAD4 heterodimer to modify transcription of TGF-β target genes
ACVR2A (P)	59	73.6	19.6	6.8	TGF-β signaling	Activin receptor type 2A; component of activin receptor; complex phosphorylates SMAD2/3
ACVR1B (P)	29	40.7	31.8	27.5	TGF-β signaling	Activin receptor type 1B; component of activin receptor; complex phosphorylates SMAD2/3
ATM	64	53.9	29.5	16.6	TGF-β signaling	Tumor suppressor; kinase that phosphorylates multiple targets including TGF-β receptor to activate TGF-β signaling and p53 to activate DNA damage pathways
ERBB2 (HER 2) (P)	38	45.6	32.5	21.9	MAPK signaling	Oncogene; EGFR; activates oncogenic Ras–Raf–MEK–ERK signaling pathway
ERBB3 (HER3) (P)	36	51.0	11.6	37.5	MAPK signaling	Oncogene; EGFR; activates oncogenic Ras–Raf–MEK–ERK signaling pathway
ERBB4 (HER4) (P)	37	42.2	40.1	17.7	MAPK signaling	Oncogene; EGFR: activates oncogenic Ras–Raf–MEK–ERK signaling pathway
KRAS (D)	173	39.3	28.7	31.9	MAPK signaling	Oncogene; component of oncogenic Ras–Raf–MEK–ERK signaling pathway
NRAS (P)	27	31.0	36.2	32.9	MAPK signaling	Oncogene; component of oncogenic Ras–Raf–MEK–ERK signaling pathway
BRAF (P)	127	71.9	21.9	6.1	MAPK signaling	Oncogene; component of oncogenic Ras–Raf–MEK–ERK signaling pathway
IGF1 (P)	4	56.6	0	43.4	PI3K signaling	Oncogene; ligand for IGF1R; Regulates cell proliferation
IGF2 (P)	4	100.0	0	0	PI3K signaling	Oncogene; ligand for IGF1R; Regulates cell proliferation
IGF1R (P)	26	72.4	12.5	15.1	PI3K signaling	Oncogene; RTK receptor phosphorylates ISR1 and ISR2
IRS1 (P)	47	44.8	14.1	41.1	PI3K signaling	Oncogene; once phosphorylated, activates PI3K-AKT/mTOR pathway and Ras–Raf–MEK–ERK signaling pathway
IRS2 (P)	15	35.0	24.8	40.2	PI3K signaling	Oncogene; once phosphorylated, activates PI3K-AKT/mTOR pathway and Ras–Raf–MEK–ERK signaling pathway
PIK3CA (P)	132	50.1	35.6	14.3	PI3K-signaling	Oncogene; triggers PI3K-Akt/mTOR pathway
PIK3R1 (P)	30	50.2	38.2	11.6	PI3K signaling	Tumor suppressor; regulates PI3CA gene
mTOR (P)	50	47.4	16.3	36.3	PI3K signaling	Oncogene; kinase activating PI3K-AKT/mTOR pathway
AKT1 (P)	11	81.3	0	18.7	PI3K signaling	Triggers PI3K-Akt/mTOR pathways that have a central regulatory role in promoting cell growth and proliferation and inhibiting apoptosis
PTEN (P)	51	51.6	28.0	20.4	PI3K signaling	Tumor suppressor; negatively regulates PI3K-ATK/mTOR pathway
TP53 (p53) (D)	320	24.2	36.3	39.5	p53 signaling	Tumor suppressor; regulates cell cycle and activates DNA damage pathways
MYC (D)	12	59.6	25.2	15.2	MYC signaling	Oncogene; codes for family of TFs

The most frequent mutations were obtained from cBioPortal and compiled according to the weighted incidence in the various sections of the colon and rectum. Key pathways associated with the genes are listed. A weighted incidence was calculated by assuming an equal representation of each section of the colon and rectum in the sample size and accounting for both the overrepresentation of proximal CRC and underrepresentation of distal and rectal CRC in the original data. Gene names followed by "D" in parenthesis have higher tendency to be mutated in distal colon cancers while those followed by "P" in parenthesis tend to be mutated in proximal colon cancers.

CRC, colorectal cancer; MMR, mismatch repair; TF, transcription factor.

Another modality through which the Wnt pathway is activated is epigenetic silencing of Wnt pathway negative regulators. Epigenetic changes, especially DNA methylation occurring at promoters that mediate gene silencing, are very early changes observed in the adenomas [61–64]. Many of the genes affected by promoter DNA methylation in cancers are already methylated in early adenomas and in the normal tissue adjacent to the cancers (field defect) [65, 66]. The negative regulator genes of the Wnt pathway that are affected by epigenetic inactivation are often important in developmental and differentiation pathways and have also been found to be methylated during aging [67–71]. As such, these additional modalities of Wnt activation, the methylation mediated inactivation of Wnt pathway negative regulators and supplementary mutations in other Wnt pathway genes aside from *APC*, may thus synergize with APC inactivation to maintain Wnt activation at multiple levels. These additional modalities of Wnt activation might be relevant in the context of the spectrum of *APC* mutations that activate the Wnt pathway to different levels [52]. This is especially relevant while considering observations that tumors with mutations in *APC* can still be responsive to Wnt ligands that suppress external Wnt signaling [72]. In the intestinal epithelial microenvironment, the antagonistic gradient of Wnt (stem-cell activating) and BMP signaling (differentiation inducing) may thus necessitate the need for multiple cell intrinsic mechanisms for Wnt pathway activation in addition to the *APC* mutation for selection of clones with stronger Wnt activation. Herein, additional mutations affecting the Wnt pathway, and especially epigenetic alterations, may provide this further impetus to early Wnt independency. Understanding the spectrum of molecular changes affecting the Wnt pathway in CRC will be critical in designing strategies for targeting its activation in cancer treatment.

### Serrated pathway

About 15%–20% of CRC cases arise through the serrated pathway wherein the precursor lesions exhibit the distinguished serrated (saw-tooth-like) morphology in the epithelial glands [73, 74]. These early lesions present as hyperplastic polyps and sessile or traditional serrated adenomas, which arise sporadically or in the context of serrated polyposis syndrome. In stark contrast to the tubular adenomas in the adenoma–carcinoma pathway, in which *APC* inactivation is a founder mutation for Wnt activation leading to the formation of the early tubular adenoma lesions, sessile serrated adenomas tend to significantly lack *APC* mutations [58, 59, 75]. Instead, mutations in *KRAS*, or more commonly *BRAF*, activate the RAS–RAF–MEK–ERK–MAPK axis as these are the earliest mutations observed in sessile serrated adenomas and its precursor hyperplastic polyps [76–84] (Figure 2). In mouse models, these mutations by themselves are not sufficient to trigger intestinal tumorigenesis, except over prolonged periods (∼1 year) in the small intestine [85] or colon [4]. Further, it has been shown that oncogenic *KRAS* and *BRAF* mutations trigger oncogene-induced senescence (OIS) via the p16-RB and ATM–ATR-mediated DNA damage response pathways [86–88]. Therefore, since OIS is one of the earliest tumor suppressor mechanisms that is triggered in response to activation of the MEK–ERK pathway by oncogenic mutations in *KRAS* or *BRAF*, founder cells have to first overcome OIS for tumor initiation to occur [85, 89, 90]. Although, in principle, oncogenic *BRAF* can induce hyperplasia, which may accompany induction of OIS [85], these hyperplastic cells continue to proliferate while accumulating further genetic and epigenetic alterations, and develop into adenomas and then progress to carcinomas [4]. In these mouse models, progression to carcinomas requires additional genetic and epigenetic changes that intensify MAPK signaling, Wnt pathway activation, and inactivation of cell-cycle and senescence pathways by epigenetic silencing of *CDKN2A/p16* [85].

Analysis of the molecular changes in the human serrated polyps has shown that early genetic and epigenetic alterations play profound roles in overcoming the OIS response and promoting early Wnt pathway activation in the absence of *APC* mutations, which forms the basis for progression of hyperplastic polyps to serrated lesions to carcinomas. One of the major regulators of OIS is the product of *CDKN2A/p16*, which is silenced by DNA methylation during progression to advanced serrated lesions [91]. The CIMP-H, which includes a high frequency of methylation in the *CDKN2A/p16* promoter, evolves during the progression from hyperplastic polyps to sessile serrated adenomas [92] (Figure 2). Analyses of individuals with serrated polyposis, i.e. incidence of multiple sessile serrated adenomas simultaneously, have identified germline alterations in genes including *ATM*, *RBL1*, *XAF1*, *PIF1*, *TELO2*, and *RNF43*, which are involved in OIS through roles in DNA repair, cell cycle, apoptosis, genome stability, and Wnt regulation [93]. Herein, inactivating mutations in *RNF43* (RING-type E3 ubiquitin ligase) is an important cancer-driver mutation, not only in the familial serrated polyposis cases [93, 94], but also in the sporadically occurring *BRAF*-mutant MSI-H CRC [20, 94–97]. Mechanistically, *RNF43* functions as a negative regulator of the Wnt pathway by ubiquitination of Wnt receptors and targeting them for degradation in colon stem cells [98]. Interestingly, although inactivation of *RNF43* may play a role in suppressing OIS induction [93], its loss in the serrated pathway is more likely to function in activating the Wnt pathway. *RNF43* mutations have been observed in *BRAF*-mutant sessile or traditional serrated adenomas, but not in their *KRAS*-mutant counterparts [99], suggesting that the alternate modalities of MEK–ERK pathway activation may determine the method by which the Wnt pathway is activated.

Furthermore, various features of the *RNF43* mutations highlight the importance of early CIMP development in driving *RNF43* mutations. This finding comes from observations that while CRCs arising in the familial serrated polyposis patients have *BRAF* mutation and are predominantly MSS, the sporadic CRC arising via the serrated pathway are predominantly MSI and CIMP-H. Particularly, the MSI-related DNA repair defects in the mismatch repair pathway are associated with development of *RNF43* mutations in the sporadic cases as the *RNF43* mutations identified in the sporadically occurring early serrated polyps and CRC derived via the serrated pathway harbor truncating mutations in mononucleotide repeats [94]. These mutations are a signature of the MSI phenotype due to loss of MLH1, which in the CIMP-H cases most frequently occurs due to DNA methylation of *MLH1* promoter [100] (Figure 2). In contrast to the sporadic serrated pathway, the familial serrated lesions with *BRAF* mutations that are MSS, i.e. without defects in the mismatch repair pathway, have been shown to harbor non-repeat tract mutations in *RNF43*. These studies have highlighted the importance of inactivating *RNF43* in the progression of the sporadic, *BRAF*-mutant, MSI-H, CIMP-H, and/or *MLH1*-methylated CRC arising via the serrated pathway. Particularly, these studies indicate that *RNF43* mutations occur via the hypermutator pathway due to silencing of *MLH1* by promoter methylation in the CIMP-H context.

### Distinct modes and roles for Wnt pathway activation in the two major CRC types

The importance of Wnt pathway activation as an early genetic dependency for progression of the serrated pathway is further highlighted by the occurrence of genetic alterations involving *RSPO* fusions in adenomas and CRC arising from the serrated pathway [94, 97, 101]. These *RSPO* fusions lead to overexpression of the secreted factor R-spondin, which amplifies Wnt signaling by negatively regulating *RNF43* and *ZNRF3*. Recent analysis of a panel of CRC-specific mutations discriminates the traditional serrated adenoma lesions from sessile serrated adenoma lesions as those that carry more frequent mutations in the Wnt pathway, interestingly indicating different dependencies on Wnt pathway mutations within the serrated pathway [99]. These studies firmly show that *BRAF*-driven CRC arising from the serrated pathway has an early dependency on Wnt activation by inactivating the RNF43–ZNRF3 axis. Therefore, mutations in components of the Wnt pathway have very early roles in the serrated pathway, similar to the role of *APC* mutations in driving the adenoma–carcinoma pathway in CRC. However, a major distinction in this parallelism is that Wnt-pathway-activating *APC* mutations have precursor roles in the adenoma–carcinoma pathway, while in the serrated pathway, the Wnt-pathway-activating mutations mentioned above most likely occur after *BRAF* mutations. In this sense, disruption of the RNF43–ZNRF3 axis in the serrated pathway may serve similar, but not identical, functions to the *APC* mutations do in the adenoma–carcinoma pathway, which may have implications for the therapeutic targeting of the Wnt pathway in different CRC subtypes.

In addition to dependency on the acquisition of mutations in *RNF43* via promoter DNA methylation mediated silencing of *MLH1*, epigenetic defects in the regulation of the Wnt and differentiation pathways may also have early and profound roles in tolerating the increased MEK–ERK signaling by oncogenic *BRAF*. For example, *CDX2* has been shown to be downregulated in CIMP-H CRC and is preferentially methylated in this CRC subtype [102–106]. In mouse models, inactivating *Cdx2* in the context of oncogenic *BRAF* (*BRAF^V600E^*, mouse-human hybrid version of the V600E oncogene) leads to serrated lesions and tumor development [107]. *Cdx2* loss in *BRAF^V600E^* inducible colon organoids allows overcoming senescence upon induction of *BRAF^V600E^* [5]. In these studies, *ex vivo* aging of mouse colon organoids involves evolution of promoter DNA hypermethylation, which facilitates activation of the Wnt pathway and predisposes transformation by oncogenic *BRAF*. Further, simultaneous inactivation of genes subject to frequent epigenetic silencing in human CIMP-H CRC, such as *CDX2*, *SFRP4*, *SOX17*, and *CDKN2A*, in freshly isolated organoids results in rapid induction of tumorigenesis upon *BRAF^V600E^*. Otherwise, in the same model, transformation takes ∼5 months after *BRAF^V600E^* induction, during which time DNA methylation alterations occur across the genome, including promoter methylation of these same genes [5]. Thus, the clinical genetics observations of CRC, and experimental observations from both *in vivo* mouse studies and *ex vivo* models, suggest that initiation of the serrated route has high reliance on epigenetic inactivation of regulators of the Wnt and senescence pathways, which allows oncogenic *BRAF* mutations to effectively drive tumorigenesis.

The key difference between the adenoma–carcinoma pathway and serrated pathway to CRC development may consequently rely on the nature of Wnt activation signals via accumulation of either mutations or sufficient epigenetic alterations by the founder cells. The extent and nature of the epigenetic alterations observed in CRC arising from these two pathways suggest different dependencies between the mutations and epigenetic alterations during CRC development. The fact that downstream activation of MEK–ERK signaling by *BRAF* shows a preference for the serrated pathway is intriguing and begs the question as to why this is the case. These insights into the molecular genetics and epigenetics of CRC development via the two major pathways, which account for the majority of CRC cases, will help refine strategies for early diagnosis and prognosis that can use a range of genetic markers, such as *RNF43*, *ZNRF3*, and *RSPO* fusions, in addition to methylation-based markers, such as *CDX2*, *CDKN2A*, and various Wnt regulator genes. Further, key differences in Wnt pathway activation could be exploited for therapeutic intervention with Wnt inhibitors that act at different levels of the Wnt pathway in combination with other targeted MEK–ERK and epigenetic inhibitors.

### Factors contributing to epigenetic changes in cells of the gastrointestinal tract

**Figure 3.**
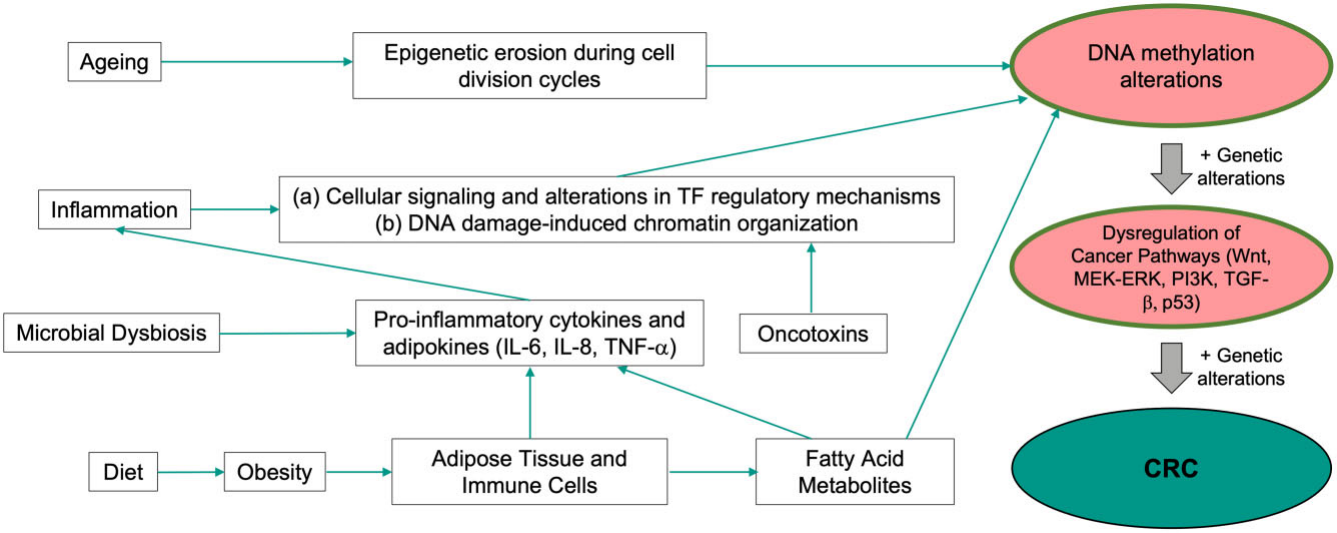
Various factors contributing to DNA methylation alterations and linked to the risk for CRC development. Various physiological and environmental risk factors (e.g. microbiome, obesity and diet, inflammation, and aging) contribute to the early acquisition of epigenetic alterations over time, eventually leading to cancer initiation following genetic driver mutations. These factors together are linked to the various macro-environmental exposures that contribute to the age-related DNA methylation changes. CRC, colorectal cancer; TF, transcription factor.

The DNA methylation alterations at promoter and other regulatory elements occur during various normal physiological processes as these epigenetic mechanisms orchestrate gene-expression changes in response to metabolic and environmental signals, including those derived from intracellular and extracellular environments. In totality, these epigenetic alterations manifest as age-related methylation changes and may reflect the cumulative impact of exposure to various environmental signals [108]. Individual exposures that directly affect the colon are important in understanding how the ensuing epigenetic changes may alter gene expression and predispose us to CRC. Below we discuss some of these factors that lead to accumulation of epigenetic changes and are known risk factors for CRC (Figure 3).

### Aging

The normal maintenance and regeneration of tissues during mammalian growth are associated with the accumulation of epigenetic changes involving both hypermethylation and hypomethylation of CpG-residues. Globally, the human genome contains ∼28 million CpG-sites unevenly distributed across the genome [109]. Of these CpG-sites, a minor subset (∼7%) are enriched in CpG-islands, which in normal cells are protected from DNA methylation [110]. About 75% of the remaining CpG-sites, scattered in the genic and non-genic regions of the genome, are normally methylated in somatic cells. During aging, substantial proportions of these non-CpG-island-associated CpG dinucleotides undergo demethylation, tending to occur at specific sites and regions of the genome [111]. These hypomethylation changes impact various aspects of genome structure and integrity, leading to deregulation in transcription and genome stability, which are important for cancer initiation and progression. On the other hand, the CpG-sites in CpG-islands, ∼50% of which are associated with gene promoter regions and otherwise normally unmethylated, gain methylation during aging [69, 112, 113]. This age-related hypermethylation occurs in CpG-sites that are associated with genes involved in development and differentiation [69, 114, 115]. Normally these genes are maintained in a repressive state by the H3K27me3 chromatin modification in progenitor cells, which in very primitive stem cells, like embryonic stem cells, co-occur with the active H3K4me3 modification termed the bivalent chromatin mark [116, 117]. Furthermore, the target sites associated with age-related hypermethylation are enriched for specific transcription factors. For example, during aging, leukocyte DNA undergoes hypermethylation at sites that are enriched for genes. These genes are predicted to be regulated by groups of transcription factors involved in biological functions related to development [118]. This phenomenon pans across species, including humans, dogs, and bats, which all show age-related methylation changes at regions related to developmental processes [118–121]. Importantly, these same regions show distinct and amplified hypermethylation in human cancers, indicating common mechanisms, and that the age-related methylation changes may induce tumorigenesis [5, 67]. The acquisition of age-related hypermethylation of multiple genes in colon cancer is important for studying colon cancer development. As mentioned earlier, methylation of genes such as *CDX2, SFRP4*, *SOX17*, *CDKN2A*, *MLH1*, and *SFRP1* are observed during normal aging [5] and are important in the development of CRC. We have shown that inactivation of some of these genes predisposes colon stem cells to tumor initiation [5]. Herein, it is possible that subclones of cells that have acquired promoter hypermethylation simultaneously in many of such genes during aging may be more prone to undergo tumorigenesis in the context of a pre-existing genetic mutation or acquired genetic mutations.

The mechanisms by which age-related hypermethylation and hypomethylation accumulate are not clear [122]. Aging involves organ growth and development until adulthood, and later organ and tissue maintenance in the context of constant tissue damage and replenishment by stem-cell divisions and tissue repair. The constant cell division in the context of environmental exposures (such as pollutants, cigarette smoke, microbial exposures including infections as well as normal flora) and physiological exposures (such as obesity and hormonal changes) is an important component of aging. Multiple studies have shown that constant cell divisions lead to global methylation changes involving genome-wide hypomethylation at intergenic and intragenic regions, along with hypermethylation at the CpG-island promoter elements [123–125]. Specifically, hypomethylation has been identified as a feature of mitotic cell division, occurring at CpG-residues in specific sequence contexts and in the late replicating DNA [125]. The replicating regions during the late S phase constitute the heterochromatic regions of the genome that are heavily methylated and replicate in large replication factories consisting of the enzyme machinery for DNA replication as well as the DNA methyltransferase (DNMT)1 [126]. DNMT1 is purported to be a catalytically slow enzyme [127] and due to the large numbers of CpG-residues in the late replicating DNA that have to be methylated during the S phase at each cell division, it is hypothesized that gradually certain residues are not faithfully methylated. Such passive loss of methylation during subsequent cell-division cycles may result in progressive hypomethylation. By contrast, regions that get hypermethylated, which are mainly in the promoter and other regulatory regions, may potentially acquire hypermethylation due to multiple cellular pathways that impinge upon transcription factor expression changes and DNA-damage-induced chromatin reorganization. The acquisition of methylation at promoter CpG-sites at these genes that are normally regulated by polycomb group proteins (primarily establishing and maintaining the H3K27me3 mark) is correlated with the number of stem-cell divisions in different organs, and accordingly can be used to determine the mitotic age of a tissue [128]. These studies have suggested that cancerous and pre-cancerous lesions can be discriminated from normal tissues by estimating the mitotic age of tissues, which tends to be higher in the former indicating increased cell divisions. Overall, current models suggest that the aging-related methylation changes arise due to inefficiencies in maintaining the epigenetic information during mitotic cycles in the context of exposures that alter chromatin and cellular signaling pathways. Various factors impacting the epigenome, discussed below, may directly fit into the broad category of such exposures to the stem-cell microenvironment that maintains tissue homeostasis.

### Microbiome

The gut microbiome represents an important microenvironmental exposure component essential in normal functioning of the colon and is also a strong modulator of CRC development (Figure 3). Distinct microbial taxonomic subgroups are altered during adenoma-to-carcinoma development [129, 130]. These studies suggest that microbial dysbiosis can occur early in the adenoma stage and that diet especially affects this dysbiosis in a manner such that consumption of red meat is associated with a hostile gut microbiome compared with the high-fiber fruits and vegetables diet. In direct relation to CRC development, individual species of bacterial populations have been shown to express pro-oncogenic genes, such as *Bacteroides fragilis* toxin (BFT), also called oncotoxins, that promote tumorigenesis [131–136]. In addition to the direct impact on CRC development by such oncotoxins, various studies have shown that the gut microbiome along with its metabolites and toxins can directly interact with epigenetic machinery and reprogram the epigenome [137, 138]. In this context, the microbiome has an important role in modulating epigenetic changes and normal postnatal development of the intestine [139]. In healthy individuals, the normal gut microbiome plays important roles in homeostasis of the intestinal epithelium by directly influencing the transcriptome and preventing inflammation [137, 139]. In contrast, dysbiosis of the gut microbiome is known to cause chronic inflammation, which could alter the epigenome and increases the risk for CRC (Figure 3). As evidence, dysbiosis in the gut due to antibiotic usage leads to an increased risk of CRC [140–142]. While the gut microbiome may cause these changes via directly affecting the immune and cytokine balance in the epithelial microenvironment [143, 144], it also reprograms the epigenome, resulting in gene-expression programs that are pro-oncogenic. Relative proportions of defined microbial species in the gut microbiota of CRC patients have been linked to methylation/demethylation changes at specific regions in the genome of epithelial cells in CRC [145]. Such roles for the CRC-associated microbiome in altering the methylation landscape of the colonic epithelial cells have been observed in fecal transplant studies, wherein the microbiota from the CRC patients when transferred to the colon of mice induced increased aberrant crypt foci and marked changes in the methylation of gene promoters in the recipient gut epithelium of the mice. Some of the genes that were identified to have altered methylation in this model are important in stem-cell functions belonging to the Wnt and Notch pathways [145].

Further, the role for microbial populations in CRC, potentially by modulating the epigenome, is indicated by studies showing that the CIMP-H CRC cases in the proximal colon are enriched in *Fusobacterium* species [146, 147]. Cancers in the proximal colon and the normal tissues from these patients have been observed to harbor distinct, invasive bacterial aggregates, called biofilms, that are associated with increased proliferation [148]. Transfer of biofilm from tumors or normal tissue of these CRC patients to *APC^Min^* mice models induces inflammation and tumor initiation [149]. In experiments comparing genome-wide expression and methylation changes in the small intestines of germ-free and/or conventional microbiota-containing mice, the presence of microbiome is associated with large changes in the epithelial transcriptional programs, of which many genes were associated with methylation changes and were enriched for processes involving intestinal epithelial proliferation and regeneration [138]. Specifically, the important tumor suppressor gene *Rb1* was observed to be downregulated in the presence of microbiome. In these studies, the tumor protective vs promoting role of the conventional microbiota is unclear as *Rb1* is downregulated. Further, the presence of the BFT has been directly associated with methylation changes in important tumor suppressors, such as *Hoxa5*, *Polg*, *Runx1*, *Runx3*, *CD37*, *Stx11*, *Tceb2*, *Lgr6*, *Cdx1*, and *Fut4*, in tumors forming in mice [150]. Interestingly, in support of the close relation between the microbiome changes associated with CRC, specific bacterial taxonomic groups, assessed by 16S rRNA sequencing, were associated with the CMS subtypes. For example, the CMS1 subgroup that is enriched for the CIMP-H subgroup is associated with increased colonization by *Fusobacterium hwasookii*, *Porphyromonas gingivalis*, *Fusobacterium nucleatum*, *Parvimonas micra*, and *Peptostreptococcus stomatis*; the CMS2 subgroup is enriched for *Selenomas* and *Prevotella* species [151]. Overall, there is building evidence for the microbiome influencing DNA methylation during normal epithelial homeostasis as well as especially during tumor development.

Molecularly, there are potentially multiple mechanisms by which a normal microbiome and conditions of dysbiosis affect the host epithelial epigenome. It has been demonstrated that bacterial dysbiosis and the oncotoxins cause inflammation and increased DNA damage. In this context, DNA damage is associated with genome-wide relocalization of epigenetic silencing complexes containing histone modifiers (polycomb repressive proteins) and DNA methyltransferases (DNMT1 and DNMT3B) at CpG-islands [152]. The recruitment of DNMT1 to the CpG-islands is directly mediated by DNA mismatch repair proteins [150, 153]. The affected CpG-islands include promoters of genes such as *SFRP4*, *MLH1*, *SFRP5*, and *SOX17*, which are known to be methylated in colon cancers and important in tumorigenesis. In addition to the methylation changes at the promoter CpG-islands, exposure to BFT causes genome-wide changes in chromatin accessibility, which is indicative of epigenetic reprogramming involving altered transcriptional programs regulated by changes in enhancer elements [154]. Further, gut microbes produce millimolar amounts of short-chain fatty acid metabolites, such as acetate, propionate, and butyrate, produced as a result of fermentation of dietary fibers [155, 156]. These metabolites are a direct source for acetyl-CoA, which is a key substrate for histone acetyl transferases, and butyrate, which is known to affect histone modifications by directly acting as an inhibitor of histone deacetylases (HDAC) that play an important role in recruiting the DNMTs to specific regions of the genome (Figure 3). In the proximal colon of mice, a Western diet, rich in fat and sucrose containing reduced amounts of fermentable dietary fibers, has reduced SCNAs and altered histone modifications compared with the regular chow diet [157]. The gut microbiome, in combination with an obesity-inducing diet, alters the histone modifications in enhancer regulatory elements of colon epithelial cells [158]. This in turn alters the expression of transcription factors, and the resulting changes in gene expression are similar to gene-expression changes during CRC development. Thus, mechanistically, the microbiome modulates the intestinal epigenome via a multitude of interactions with the host tissue, and the key challenges are to determine the epigenome modulating roles of the individual components, such as the direct effect of oncotoxins, interactions with the epithelial and immune components, inflammation, and the role of microbial metabolites. A cumulative understanding of how the ensuing epigenetic changes impact colon stem-cell maintenance and differentiation is important to understand CRC risk.

### Metabolic influences: obesity and diet

Obesity is one of the major risk factors for colon cancers [159–161]. Adipose tissue is enriched in immune cells such as macrophages and secretes pro-inflammatory adipokines (e.g. tumor necrosis factor [TNF]), which results in chronic low-grade systemic inflammation [160, 162]. Several studies have shown that obesity directly influences gene-expression changes with important roles in predisposing to tumorigenesis. Most direct analyses on the effect of obesity on epigenetic alterations come from studies in mice, wherein it has been shown that obesity is directly associated with an increase in pro-oncogenic signals, such as RAS, PI3K, and JNK signaling pathways. Changes in these pathways are brought about by alterations in the histone H3K27-acetylation (H3K27ac) mark at enhancer regulatory regions in the genome, which is an important indicator of active enhancer regulatory elements that control expression of target genes from a distance [163]. Obesity induces significant loss of H3K27ac at enhancer regions that are specific to colon tissue, and the loss of activity of these enhancer elements is similar to that observed in colon cancers [163, 164]. The transcriptomic and epigenomic alterations may result from the direct impact of diet as well as due to obesity-induced metabolic and inflammatory changes. Diet is known to affect metabolism and the availability of different species of long-chain fatty acids, and the latter may directly interact with cell signaling and epigenetic machineries. For example, butyrate is an important product of metabolizing a low-fat diet, which in addition to being an energy resource is also a known modulator of HDAC. Further, obesity-inducing high-fat diets in mice stimulate an increase in Lgr5+ colon stem cells and stem-cell function, via activation of PPAR-δ target genes, which are involved in the oxidation of long-chain fatty acids [165]. Mice fed with an obesity-inducing diet primarily show significant changes in expression of genes involved in metabolic processes, such as lipid metabolism, carbohydrate metabolism, and energy production, and importantly these genes show similar upregulation or downregulation in human CRC. Administration of a high-fat diet involves a shift from butyrate-dependent metabolism, which inhibits intestinal stem/progenitor cell proliferation, to metabolism of the stem cell promoting long-chain fatty acid [166]. Importantly, these studies have shown that, in parallel with metabolic changes, a high-fat diet induces gains and losses of DNA methylation at regulatory elements throughout the genome. These studies have reported that the majority of DNA methylation gains and losses are associated with enhancer regulatory elements, which potentially control cancer-related metabolic genes. Over the long term, these methylation changes are of important consequence as they impact future gene-expression changes. Thus, obesity and high-fat diet are associated with alterations in both histone and DNA methylation changes with important consequences for increasing risk of colon cancer. The mechanisms by which obesity alters DNA methylation are not clear and, as mentioned earlier, may involve direct impact on epigenetic enzymes by the metabolites of fatty acid metabolism or due to inflammatory signals induced by the obesity-associated adipose tissues. In regard to the latter, adipocytes release a number of hormones, as well as attract immune cells that may create an inflammatory environment resulting in high levels of pro-inflammatory cytokines, such as IL-6, IL-1β, and TNF-α, released by the adipocytes as well as the infiltrating immune cells [162]. Such alterations of the inflammatory environment linked to obesity can impact the epigenome, as discussed further in the inflammation section (Figure 3).

### Inflammatory microenvironment

Inflammation can occur on its own, due to the gut microbiome, or due to diet and obesity, and it is a very strongly associated factor with the cancer microenvironment. The relationship between inflammation and CRC has been shown to be reciprocal in nature as chronic inflammation increases the risk of cancer initiation and cancer progression often leads to an inflamed microenvironment [167]. Accordingly inflammatory genes, such as IL-1, IL-6, IL-17A, and IL-23, are increased in most sporadic CRC cases [168]. Looking more closely at the relationship between inflammation and CRC, it is well known that patients with chronic inflammation in their bowels are at a higher risk for CRC. Diseases that result in inflammation of the bowels are majorly inflammatory bowel diseases (IBD) such as Crohn’s disease and ulcerative colitis. Meta-analysis of population-based cohort studies has shown that the incidence of CRC is positively correlated with worse IBD severity and longer disease duration with cumulative risks of CRC at 1%, 2%, and 5% after ≤10, ≤20, and >20 years of IBD diagnosis, respectively [169]. Further, obesity also results in chronic low-grade inflammation as well as alterations in the gut microbiome as mentioned above. Chronic inflammation has been shown to speed up the rate of age-related epigenetic changes via aberrant DNA methylation patterns seen in sporadic CRC [170]. A temporal analysis of methylation patterns based on the infection-associated inflammatory response to *Helicobacter pylori* shows that several inflammation-related genes, such as *CXCL2*, *IL-1b*, *NOS2*, and *TNF-α*, positively correlate with temporal changes in methylation levels. Further, when *H. pylori* is administered but the infection-associated inflammatory response is suppressed using the immunosuppressive drug, cyclosporin A, alterations in DNA methylation no longer occur, implying that inflammation itself is inducing the aberrant methylation changes [171].

One mechanism for these aberrant methylation changes may be due to higher expression levels of DNMT1, the enzyme that methylates and silences genes, in inflamed mucosa. Treatment of colon cancer cells with the inflammatory biomarker IL-6 alone results in an increase in DNMT1 expression independently of *de novo* gene expression as well as an increase in methylation of the promoter regions of tumor suppressor genes, leading to their downregulation. This downregulation in expression can be prevented by pre-incubation with a DNMT1 inhibitor, showing that inflammation-associated changes in DNA methylation patterns may occur through the activity of DNMT1 [172]. Studies have suggested that the mechanism by which inflammation promotes CRC may occur through the methylation of specific genes. In particular, the inflammatory NF-κB and STAT3 signaling pathways play an important role by secreting a large number of pro-inflammatory cytokines into the cell microenvironment (Figure 3). These pro-inflammatory cytokines include IL-6 and TNF, which aid in the methylation of distinct tumor suppressor genes including *ITGA4*, *TFPI2*, *VIM*, *SOCS3*, *p14^ARF^*, *p16^INK4A^*, and *PTX3* that promote malignant transformation [168].

The cytochrome P450s *CYP2E1* and *CYP1B1* along with *FXR* and *VDR* are also associated with inflammatory conditions and deficiency via methylation, which may allow weakened defenses against carcinogens, resulting in carcinogenesis [167]. Inflammation-induced epigenetic silencing of these genes typically occurs by either of two mechanisms. The first mechanism is hypermethylation of CpG-island promoters, which results in the CIMP. Comparing the methylation rates for seven CpG-island sites in mucosae of individuals who are healthy and individuals infected by *H. pylori* inducing inflammation, Maekita *et al*. [173] show that infection significantly increases methylation of CpG-islands to various levels and that these methylation levels of specific CpG-islands were predictive of cancer risk in health individuals. The second mechanism of methylation is a novel epigenetic program identified by Abu-Remaileh *et al*. [174] using monoalkyl methylation maps of a colitis-induced mouse colon cancer model. This program is characterized by hypermethylation of DNA methylation valleys (DMVs), which leads to epigenetic silencing of DMV-associated genes. Ultimately, inflammation-induced epigenetic silencing via hypermethylation of CpG-island promoters or DMVs aids in the malignant transformation of inflamed mucosa to CRC. Lastly, inflammation has also been shown to promote early epigenetic modifications through histone modification, microRNAs, and lncRNAs; however, we have chosen to focus on DNA methylation specifically for this review.

## Conclusion

The long debate as to what constitutes the early dependencies for CRC development is still ongoing. It is becoming clear that the modes of Wnt pathway activation may rely on different mechanisms in the two major pathways of CRC. Although epigenetic changes seem to play important roles in the classical pathway, the serrated pathway appears to be more dependent on early epigenetic changes. Understanding how an array of environmental and physiological factors affect DNA methylation in colon epithelial cells, and defining the gene promoter and regulatory elements involved, will be important in understanding and preventing the risk for CRC in the future.

## Authors’ Contributions

S.P. and H.E. conceived and wrote the manuscript. Both authors read and approved the final manuscript.

## Funding

This work was supported by the National Cancer Institute and National Institute on Aging at the National Institutes of Health [NIH/NCI R01CA229240; NIH U01AG066101].
